# Systematic Review: The Impact of COVID-19 Vaccination on Myocarditis Risk and Recovery

**DOI:** 10.3390/clinpract16040077

**Published:** 2026-04-17

**Authors:** Yibo Liu, Christopher Khatchadourian, Luke Sanders, Quincy Eweroke, Cyvannah Warner-McCutcheon, Jackson Lewis, Joaquin Santos, Vishwanath Venketaraman

**Affiliations:** 1 Department of Biomedical Sciences, Kansas College of Osteopathic Medicine, Wichita, KS 67202, USAlsanders2@mail.kansashsc.org (L.S.); qeweroke@mail.kansashsc.org (Q.E.); cwarnermccutcheon@mail.kansashsc.org (C.W.-M.); jlewis5@mail.kansashsc.org (J.L.); jsantos1@kansashsc.org (J.S.); 2 Department of Pathology, University of Southern California, Los Angeles, CA 90033, USA; christopher.khatchadourian@med.usc.edu; 3 Los Angeles General Medical Center, Department of Pathology, Los Angeles, CA 90033, USA; 4 College of Osteopathic Medicine of the Pacific, Western University of Health Sciences, Pomona, CA 91766, USA

**Keywords:** COVID-19, vaccination, myocarditis, Pfizer, Moderna, coronavirus

## Abstract

**Background:** Myocarditis is an uncommon but recognized adverse event following mRNA COVID-19 vaccination, with risk varying by age, sex, dose number, and vaccine product. Clarifying the magnitude of risk, clinical course, and recovery—relative to myocarditis following SARS-CoV-2 infection—is essential for risk–benefit assessment and public health guidance. **Methods:** We performed a systematic PubMed and Embase search (January 2020–December 2024) and synthesized cohort, registry, and surveillance data on myocarditis incidence and outcomes following mRNA COVID-19 vaccination. Outcomes included incidence, observed-to-expected (OE) or incidence rate (IRRs) ratios, hospitalization, and short-term recovery. Study selection followed PRISMA 2020 systematic review guidelines. **Results:** Myocarditis following mRNA COVID-19 vaccination was identified as a rare adverse event, most commonly occurring after the second dose and in younger male individuals. Across multiple cohort and registry-based studies, cases were generally mild and self-limited, with most patients recovering without complication. In contrast, myocarditis following SARS-CoV-2 infection was consistently associated with more severe outcomes, including higher rates of hospitalization and mortality. **Conclusions:** Vaccine-associated myocarditis is rare, typically mild, and self-limited, with excellent short-term recovery; vaccinated individuals also exhibit lower odds of in-hospital death and intubation. In contrast, infection-associated myocarditis is more frequent and severe. Overall, the benefit–risk profile of mRNA vaccination remains strongly favorable.

## 1. Introduction

Myocarditis is an inflammatory disease of the myocardium characterized by myocyte injury, necrosis, and immune-cell infiltration that can impair cardiac function and electrical stability. Etiologies are diverse and include viral infection, as well as autoimmune disorders, hypersensitivity reactions, and drug- or toxin-induced injury [1,2]. The disease can range from asymptomatic subclinical inflammation to fulminant heart failure, and chronic cases may progress to dilated cardiomyopathy or arrhythmic death, underscoring its cardiovascular importance. Pathophysiologically, myocarditis involves cytotoxic T-cell activation, cytokine release, and, in some cases, direct viral or toxic myocyte injury that leads to myocardial edema and impaired ventricular function [1].

Since the onset of the Coronavirus Disease 2019 (COVID-19) pandemic, Severe acute respiratory syndrome coronavirus 2 (SARS-CoV-2) has been recognized as a potent trigger of myocarditis. Mechanistic evidence indicates that myocardial injury may result from direct viral invasion through angiotensin-converting enzyme 2 (ACE2) receptors, immune-mediated inflammation, and microvascular thrombosis [1]. Population-based studies during the pandemic demonstrated that infection-related myocarditis frequently presents as a severe, systemic inflammatory process associated with increased morbidity and mortality [1]. As vaccination campaigns expanded globally, reports began to document cases of myocarditis temporally associated with mRNA-based vaccines, most notably among adolescent and young adult males following the second dose [3,4,5].

Large-scale population studies confirmed this temporal pattern. In a Nordic cohort of 23 million residents, the incidence-rate ratios were approximately fivefold for BNT162b2 (Pfizer-BioNTech) and nearly fourteenfold for mRNA-1273 (Moderna) among men aged 16–24 years [3]. Similarly, observed-to-expected rate ratios were approximately 15 overall within seven days post vaccination [4]. Despite these elevated relative risks, the absolute incidence remains very low—roughly one to three cases per 100,000 administered doses—and most affected individuals experience a complete recovery [1]. In the studied cohort of children aged 5–11 years, no cases of myocarditis were observed, underscoring the rarity of this event in younger age groups [6].

Recognition of vaccine-associated myocarditis has prompted important public-health discussions about balancing the benefits of COVID-19 vaccination against its potential risks. Although mRNA vaccines carry a small and typically transient risk of myocarditis, they provide substantial protection against severe COVID-19 [7]. Cohort analysis indicates that for every million second doses administered, vaccination prevents thousands of infections and hundreds of hospitalizations while contributing only a small number of generally mild myocarditis cases [4]. Distinguishing this short-lived, reversible inflammation from the more severe myocarditis caused by SARS-CoV-2 infection remains essential for clinical communication and sustaining public confidence. Ongoing research—particularly studies employing standardized diagnostic criteria and extended follow-up—is needed to further elucidate the mechanisms, outcomes, and demographic risk factors for both infection- and vaccine-associated myocarditis [5,7]. Our review aims to compare the incidence and outcomes of myocarditis following COVID-19 infection and mRNA COVID-19 vaccination.

## 2. Materials and Methods

### 2.1. Search Strategy

A systematic search using the PubMed and Embase databases was performed. The search was limited to studies published between January 2020 and December 2024. The array of search terms used was as follows: (“myocarditis”) AND (“COVID-19 Vaccine” OR “mRNA vaccine” OR “BNT162b2” OR “mRNA-1273”) AND (“risk” OR “incidence” OR “outcome”). The following filters were applied to the search terms when applicable: studies between 2020 and 2024, involving human subjects, published in the English language, and using mRNA vaccines. Reference lists from major publications were manually reviewed to ensure completeness and capture of related cohort data.

### 2.2. Inclusion Criteria

• Cohort studies
• Observational studies
• Randomized controlled studies

### 2.3. Exclusion Criteria

• Review papers, Systematic reviews, and Meta-analyses were excluded to prevent reporting bias
• Open label trials (non-randomized trials) to prevent the placebo effect
• Studies without individual data on myocarditis rates following COVID-19 mRNA vaccination
• Case reports and Case series
• Small studies (<101 participants)
• Case–Control studies, studies with vaccination as the measured outcome, or studies with myocarditis as the exposure are excluded to prevent incorrect representation of Myocarditis incidence from COVID-19 vaccination
• Studies primarily involving non-mRNA or non-COVID-19 vaccines unless the studies’ primary findings or analyses addressed myocarditis associated with mRNA COVID-19 vaccines
• Studies with no results published
• Surveys and interviews to prevent recall bias
• Cross-sectional studies

### 2.4. Analytical Approach

**Figure 1:**
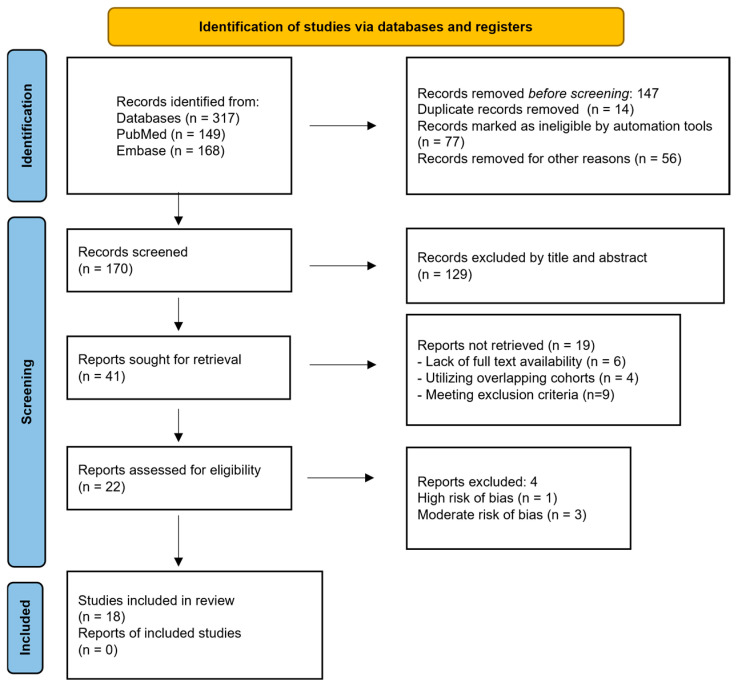
Flow diagram illustrating the systematic review selection process. Scheme 2021. N71. Doi: 10.1136/bmj.n71 [8] (accessed on 21 February 2026). This work is licensed under CC BY 4.0. To view a copy of this license, visit https://creativecommons.org/licenses/by/4.0/.

Articles were filtered post-search as demonstrated in Figure 1. We extracted and standardized data on myocarditis incidence, hospitalization, and outcomes in vaccinated or infected individuals. Comparative analysis emphasized severity, mortality, and recovery differences between vaccine-associated and infection-associated myocarditis. Study selection followed Preferred Reporting Items for Systematic reviews and Meta-Analyses (PRISMA) 2020 guidelines (Supplementary Materials, File S1), and Table 1 summarizes the included studies.

**Table 1:** Summary of included studies.

Author (Year)	Country	Study Design	Population (N)	Vaccine Type	Dose	Outcome Measured	Incidence/Results	Effect Size (RR/OR/HR)	*p*-Value	Key Findings
Corrao et al. (2022) [9]	Italy	Cohort	9,184,146	Pfizer, Moderna	1–2	Myocarditis; severe COVID outcomes	124 vs. 463 cases (9.9 vs. 5.2 per million person-months)	Moderna HR up to 5.5; Pfizer HR 1.5	NR	Increased risk (Moderna > Pfizer), but rare and non-fatal
Bots et al. (2022) [10]	Europe	Cohort + SCRI	≈35 million	Pfizer, Moderna	1–2	Myocarditis	Higher risk after dose 2	IRR up to 7.8 (Pfizer); 6.1 (Moderna)	NR	Rare, mild, higher risk in <30 years
Naveed et al. (2022) [4]	Canada	Cohort	NR	Pfizer, Moderna	1–2	Myocarditis	299 vs. 59 per million (Moderna vs. Pfizer)	NR	NR	Higher observed vs. expected rates; mild, no deaths
Simone et al. (2022) [11]	Multicenter	Cohort	NR	Pfizer, Moderna	3	Myocarditis	Rare; lower than dose 2	NR	NR	Mild, self-limited, no deaths
Nygaard et al. (2022) [12]	Denmark	Cohort	208,088	Pfizer	1–2	Myopericarditis	≈4–5 per million	RR 4.6 (wide CI)	0.005 *	Extremely rare; mild; no ICU or deaths
Moreira, Jr. et al. (2022) [13]	Global	Randomized controlled trial	≈10,000	Pfizer	3	Myocarditis; COVID outcomes	0 myocarditis cases	NR	NR	Strong protection; no myocarditis observed
Karlstad et al. (2022) [3]	Nordic countries	Cohort	23 million	Pfizer, Moderna	1–2	Myocarditis	≈5–6/100,000 (Pfizer); ≈18–19/100,000 (Moderna)	IRR 2.28 (Pfizer); 6.47 (Moderna)	NR	Highest risk in young males; rare and mild
Levi et al. (2023) [14]	NR	Prospective Cohort	NR	Pfizer	4	Myocardial Injury	No myocarditis; transient troponin elevation	NR	NR	No clinically significant myocardial injury
Nordstrom et al. (2023) [15]	Sweden	Retrospective Cohort	832,273 adolescents	Pfizer, Moderna	≥1 dose	Myocarditis; serious adverse events	86 vs. 19 cases (0.57 vs. 0.43 per million person-days)	Adjusted HR 0.99 (0.60–1.65)	0.98	No increased risk; rare and similar between groups
Yechezkel et al. (2023) [16]	Israel	Cohort	≈100,000	Pfizer	4	Myocarditis	0 cases	NR	NR	No myocarditis after fourth dose
DeSilva et al. (2023) [17]	USA	Cohort	≈80,000	Pfizer, Moderna	Booster	AESIs	No myocarditis signal	NR	NR	Reassuring safety in pregnancy
Wan et al. (2024) [18]	Hong Kong	Comparative Cohort	639,000–1.8 million	Pfizer, CoronaVac	2–3	Myocarditis	Higher incidence with Pfizer after 2 doses	IRR 8.999 (1.14–71.02)	NR	Signal present after 2 doses; not significant after 3
Figueroa et al. (2024) [19]	NR	Randomized control trial	≈3700	Moderna	1–2	Myocarditis	1 nonserious case	NR	NR	Rare; resolved quickly
Sritharan et al. (2024) [20]	Australia	Cohort	≈1714	Pfizer, Moderna, AstraZeneca	NR	Cardiovascular outcomes	Not myocarditis- focused	NR	NR	Weak fit; not vaccine myocarditis study
Faksova et al. (2024) [7]	Multinational	Cohort	99 million	Pfizer, Moderna, AstraZeneca	Multiple	AESIs (Including Myocarditis)	Elevated observed-to-expected ratios	NR	NR	Confirms myocarditis as rare safety signal
Oda et al. (2024) [21]	Japan	Randomized control trial	828	ARCT-154, Pfizer	4	Safety	No myocarditis cases	NR	NR	No signal; booster safety supported
Bianchi et al. (2024) [22]	Italy	Cohort	2.9 million doses	Combined group of mRNA vaccines	2	Myocarditis	17.9 per million	aOR 0.4 (0.3–0.5)	NR	Suggests no causal increase; interpret cautiously
Andrews et al. (2024) [23]	England	Cohort (preprint)	NR	Pfizer, Sanofi	Booster	Safety	Not myocarditis focused	NR	NR	Not myocarditis focused

Key: AESI, adverse event of special interest; aOR, adjusted odds ratio; CI, confidence interval; HR, hazard ratio; OR, odds ratio; RR, relative risk; SCRI, self-controlled risk interval; SD, standard deviation; IQR, interquartile range; NR, not reported; *, statistically significant result.

### 2.5. Studies with Similar Cohorts

The methods section of each article was interrogated to determine what database was used for the identification of subjects. If similar databases were used between studies, the types of subjects pooled from the database were assessed for overlap. If the different studies used the same database but different sub-populations (e.g., Paper 1 only studies adolescents from database X, while Paper 2 only studies the elderly from database X), then both papers were included during the screening process. If there was overlap in the database and sub-population between studies, a risk of bias assessment was used to pick the single highest-quality study between them to include in the systematic review.

### 2.6. Risk of Bias Assessment

• Four authors assisted in the risk of bias assessments (ROBA) for each study.
• The following ROBAs were used according to each type of study:
  ○ Newcastle-Ottawa scale was used for the assessment of Cohort studies
  ○ Risk Of Bias in Non-randomized Studies—of Exposure (ROBINS-E) was used for the assessment of non-randomized studies of exposures
  ○ Risk-of-bias tool for randomized trials (RoB 2) was used for the assessment of randomized controlled trials
• Conflicting results between the authors were handled as follows:
  ○ Overall risk of bias and quality were the primary comparison measures
  ○ If there were discrepancies between the author’s ratings of individual domains used in the calculation of overall risk, but the overall risk of bias ratings was identical, no further resolution was warranted, and the overall risk of bias was recorded as concordant between authors.
• If there were discrepancies resulting in differences in the overall risk of bias assessment, they were resolved by discussion between the authors and a final overall risk of bias was assigned to the study.

## 3. Results

### 3.1. Overall Incidence and Demographic Patterns

Across multiple large cohort studies, myocarditis following mRNA COVID-19 vaccination remained a rare adverse event but occurred more frequently in younger male individuals, particularly after the second dose. In the Nordic cohort of 23 million residents, the highest risk was observed in males aged 16–24 years after dose two, with incidence rate ratios of 2.28 for Pfizer and 6.47 for Moderna. Similarly, Ontario surveillance data showed markedly higher reporting rates after Moderna dose two compared with Pfizer dose two in males aged 18–24 years. These findings support a consistent demographic pattern of rare overall incidence with higher risk concentrated in young males after the second dose [7,22,24].

### 3.2. Comparison of Vaccine Products

Comparative analyses across multiple cohort studies demonstrate a higher incidence of myocarditis associated with Moderna compared to Pfizer, particularly after the second dose. In a large population-based study from Lombardy, adjusted hazard ratios reached 5.5 following Moderna dose two compared to approximately 1.5 for Pfizer. Similarly, multinational and registry-based studies consistently showed greater risk with Moderna, especially in younger male populations. These findings highlight the importance of vaccine type and dosing when evaluating myocarditis risk [7,22].

### 3.3. Clinical Course and Outcomes

Clinical presentation of post-vaccination myocarditis most commonly included chest pain, dyspnea, and palpitations. Across multiple cohort studies, the majority of cases were mild and self-limited, with short hospital stays and favorable outcomes. Most patients recovered fully without complications, with preserved or only mildly reduced cardiac function. ICU admissions were rare, and no deaths were reported in vaccinated individuals. These findings consistently demonstrate that vaccine-associated myocarditis follows a generally benign clinical course [17,22,25].

### 3.4. Special Populations

Available data on special populations remain limited; however, existing studies suggest that myocarditis following mRNA vaccination is extremely rare among children, particularly those aged 5–11 years, with no statistically significant increase in risk observed. When cases occurred, they were mild and self-limited. In adolescents and young adults, incidence rates were higher, especially among males, consistent with broader population trends. Data on pregnant individuals and those with comorbidities remain limited, but current evidence does not indicate a significantly increased risk in these groups [17,19,22].

### 3.5. Comparison with SARS-CoV-2 Associated Myocarditis

Compared to vaccine-associated myocarditis, SARS-CoV-2-associated myocarditis is associated with significantly worse clinical outcomes. Multiple studies have demonstrated higher rates of hospitalization, intensive care unit admission, and mortality among patients with COVID-19-related myocarditis. In contrast, myocarditis following mRNA vaccination is typically mild, self-limited, and associated with favorable recovery in the majority of cases, with rare ICU admissions and no reported mortality in most cohorts. These findings highlight a clear clinical distinction and support the overall favorable risk–benefit profile of mRNA vaccination [20,22,25].

## 4. Discussion

### 4.1. Interpretation of Findings

Across multinational studies, mRNA vaccine-associated myocarditis is rare, generally mild, and self-limiting, with highest risk in the young male demographic (16–24 years) and a lower incidence in combination with milder symptoms in females, children, and older adults [3,6]. Additionally, no increased risk of myocarditis was noted in pregnant women [17]. Ilonze et al. and Truong et al. further confirmed that vaccine-associated myocarditis typically presents with acute chest pain, transient troponin elevation, and rapid clinical recovery, contrasting with COVID-19 infection-induced myocarditis, which is more severe and prolonged [5].

### 4.2. Risk–Benefit Evaluation

Although Faksova et al. identified a slightly elevated myocarditis rate post-mRNA vaccination, the absolute risk remains extremely low (≈0.001%) [7]. Bianchi et al. reported a protective association between vaccination and myocarditis risk, particularly among young adults, while Sritharan et al. demonstrated that vaccinated patients had lower hospitalization and mortality rates after COVID-19 infection [20,22].

The substantially lower morbidity and mortality among vaccinated individuals, combined with the protective benefits of vaccination against severe COVID-19 highlight that the benefit–risk balance remains strongly favorable.

### 4.3. Limitations

This analysis is subject to several important limitations inherent to the current literature on myocarditis following SARS-CoV-2 infection and mRNA vaccination. The literature search was limited to two databases (PubMed and Embase), which may have resulted in the omission of relevant studies indexed in other sources (Scopus and Cochrane Library, for example).

Most available data were derived from large observational and registry-based studies, which, while valuable for population-level assessment, are limited by their nonrandomized and retrospective design [3,4]. These investigations frequently rely on administrative databases and passive surveillance systems such as Vaccine Adverse Event Reporting System (VAERS) and national hospital registries, which are susceptible to underreporting, coding errors, and incomplete clinical documentation [22]. Despite efforts to exclude overlapping data, the possibility of duplicate contributions from the same registries cannot be completely eliminated. In both the Nordic and Canadian population studies, myocarditis cases were identified through hospital discharge diagnoses recorded in national and provincial registries—using ICD-10 codes in Canada and comparable registry-based diagnostic coding in the Nordic countries—rather than standardized clinical adjudication, which may contribute to over or underestimation of incidence [3,4]. Additionally, mild or subclinical cases that did not necessitate hospitalization were often excluded, emphasizing more severe presentations and underrepresenting the broader spectrum of post-vaccination myocarditis [6].

Another major limitation involves variability in diagnostic criteria across studies, leading to high heterogeneity in design, populations, definitions of myocarditis, and follow-up periods, limiting the possibility of direct comparisons and pooled analysis. Some investigations defined myocarditis solely through elevated cardiac biomarkers or nonspecific imaging findings, while others required cardiac MRI confirmation or biopsy-proven inflammation [1,2]. The histopathologic findings in post-vaccine myocarditis range from lymphocytic inflammation to microvascular thrombosis, but biopsy confirmation remains uncommon, limiting mechanistic certainty [1]. Additionally, a healthy vaccine effect cannot be entirely ruled out, whereby individuals who choose to be vaccinated may have an overall more favorable health profile.

Finally, limited long-term follow-up continues to constrain understanding of chronic cardiac outcomes after both vaccine- and infection-associated myocarditis. Most population studies have evaluated outcomes within 7 to 28 days after vaccination or infection, with few extending observation beyond three months [3,4]. Consequently, the potential risks of persistent myocardial fibrosis, arrhythmias, or recurrent inflammation remain uncertain [1]. Longitudinal imaging and recovery data are especially limited in adolescents and young adults, the demographic at greatest risk for acute myocarditis events [2,6]. Recent reviews have emphasized the importance of large, prospective, multicenter studies to evaluate long-term cardiac recovery, fibrosis burden, and overall prognosis following vaccine- and infection-related myocarditis [22,26,27]. Until such data become available, interpretations of chronic risk should remain cautious and considered within the context of the overall rarity and typically mild short-term course of vaccine-associated myocarditis.

## 5. Conclusions

Myocarditis following mRNA COVID-19 vaccination is uncommon, transient, and has a favorable prognosis, with the majority of cases resolving fully with conservative therapy. In contrast, COVID-19 infection-related myocarditis is more frequent, clinically severe, and associated with higher morbidity and mortality. Data from multiple global cohorts, including over 100 million vaccinated individuals, consistently show that mRNA vaccination significantly reduces the risk of cardiovascular complications, hospitalization, and death. Furthermore, the safety profile in pediatric, female, and pregnant populations remains excellent, supporting continued global use of mRNA vaccines as a key tool in pandemic control and cardiovascular protection.

## 6. Future Research Directions

Future research should focus on clarifying the long-term cardiac outcomes and underlying mechanisms of myocarditis following mRNA COVID-19 vaccination. Although current evidence shows that most cases are mild and self-limited, longitudinal follow-up using cardiac MRI and biomarkers is needed to assess potential subclinical fibrosis, diastolic dysfunction, or arrhythmogenic remodeling. Mechanistic studies comparing vaccine-induced and infection-induced myocarditis through immunophenotyping and genomic analyses could reveal specific immune signatures, hormonal influences, or genetic susceptibilities such as HLA or ACE2 variants that predispose individuals to adverse cardiac inflammation. Developing predictive risk models integrating demographic, hormonal, and clinical variables may guide personalized vaccination strategies for higher-risk groups, particularly young males.

Additionally, future vaccine optimization—through adjusted dosing, extended intervals, or next-generation platforms like protein-subunit or self-amplifying mRNA vaccines—should aim to maintain immunogenicity while minimizing myocarditis risk. Continued evaluation of booster and heterologous vaccination strategies is also warranted to determine their long-term cardiovascular safety and protective efficacy. Finally, ongoing safety surveillance in pediatric and pregnant populations, coupled with international registry collaboration, will be critical to confirming vaccine safety across all demographics and ensuring evidence-based updates to global immunization policies.

## Data Availability

No new data was created in the production of this literature review. The data presented in this study was derived from the following resources available in the public domain(s) of: https://pubmed.ncbi.nlm.nih.gov/ (accessed on 14 January 2026); https://www.embase.com/landing?status=grey (accessed on 5 March 2026).

## Supplementary Materials

The following supporting information can be downloaded at: https://www.mdpi.com/article/10.3390/clinpract16040077/s1. File S1: PRISMA 2020 checklist.

## Abbreviations

The following abbreviations are used in this manuscript:

ACE2	Angiotensin-converting enzyme 2
VAERS	V-safe and vaccine adverse event reporting system
MIS-C	Multisystem inflammatory syndrome in children
OE	Observed-to-expected
